# Supplementary material for: CarRS Two-Component System Essential for Polymyxin B Resistance of Vibrio vulnificus Responds to Multiple Host Environmental Signals
Source: Microbiol Spectr. 2023 Jun 8;11(4):e00305-23. doi: 10.1128/spectrum.00305-23 (PMC10433830; doi:10.1128/spectrum.00305-23)
Supplement: Supplemental file 1 — Fig. S1 to S2 and Tables S1 to S3. Download spectrum.00305-23-s0001.docx, DOCX file, 0.8 MB [file spectrum.00305-23-s0001.docx]

**
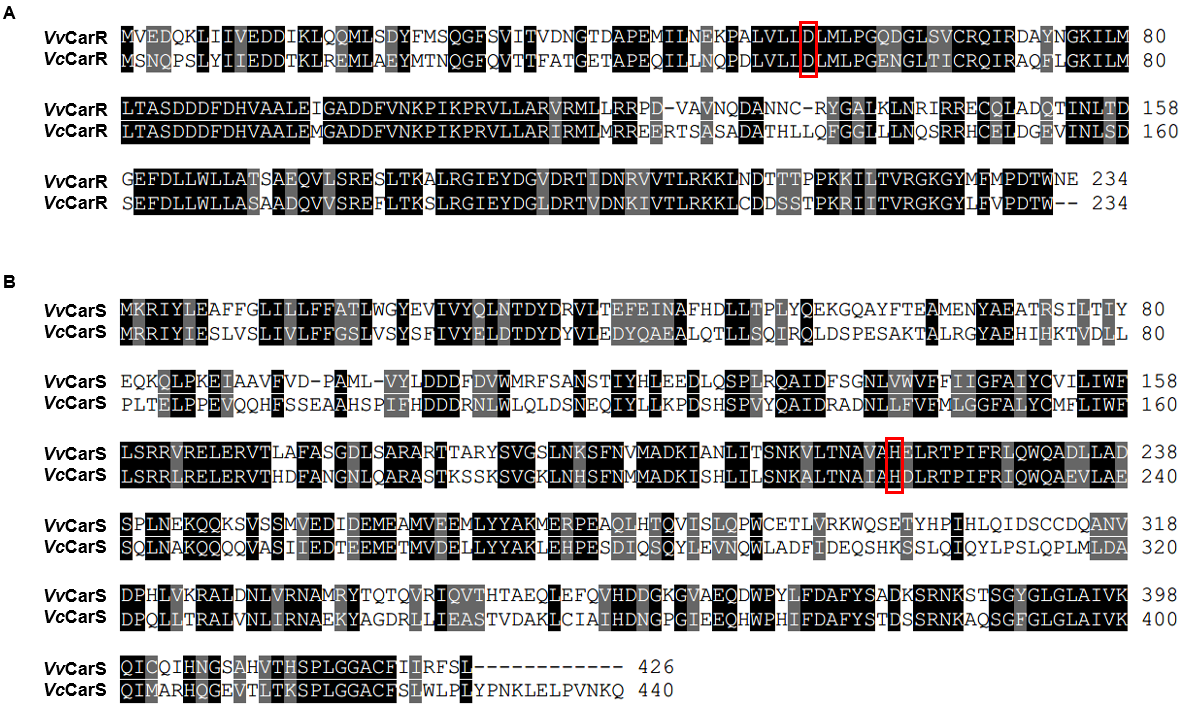
Figure S1. Sequence analysis of *V. vulnificus* CarRS and *V. cholerae* CarRS.** The amino acid sequences of *Vibrio* CarR (A) and CarS (B) were retrieved from the NCBI protein database and aligned using the T-Coffee alignment program (1). Identical and similar sequences are shaded in black and gray, respectively. Missing sequences are indicated by dashed lines. Conserved Asp and His residues putatively involved in phosphorylation of CarR and CarS, respectively, are indicated by red boxes. *Vv*CarR, *V. vulnificus* CarR; *Vc*CarR, *V. cholerae* CarR; *Vv*CarS, *V. vulnificus* CarS; *Vc*CarS, *V. cholerae* CarS.

**
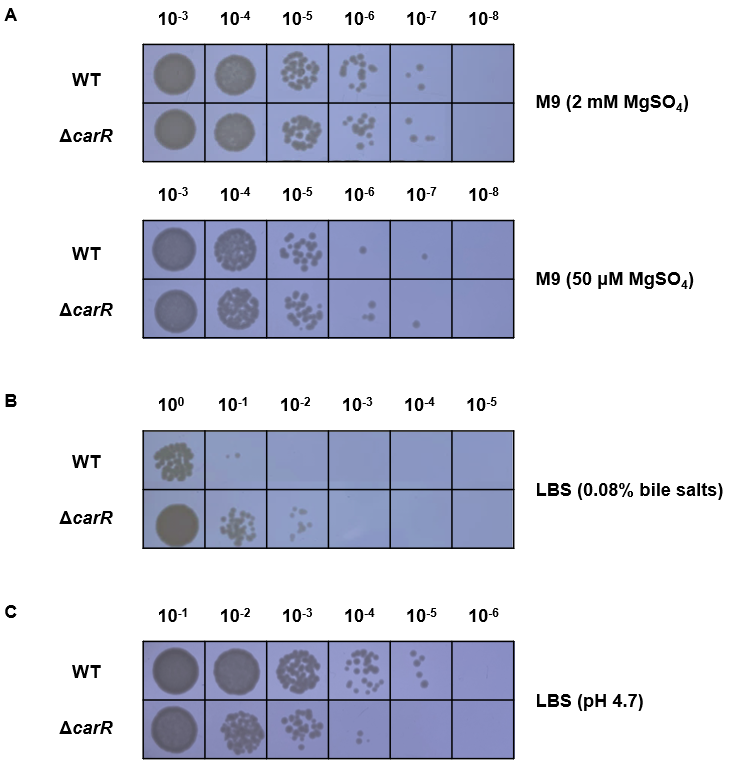
**

**Figure S2. Effect of CarR on growth of *V. vulnificus* under host environmental stresses.** The *V. vulnificus* strains were incubated in M9 minimal medium containing 2 mM or 50 μM MgSO_4_ for 9 h (A), in LBS containing 0.08% (w/v) bile salts for 2 h (B), or in LBS adjusted to pH 4.7 for 2 h (C). Then, the *V. vulnificus* cultures were serially 10-fold diluted and spotted on the LBS agar plates. After 14 h incubation, the plates were photographed. WT, wild type; Δ*carR*, *carR* mutant.

**Table S1. The genes differentially expressed by the *carR* deletion**

| Locus tag^a^ | Fold change (log_2_)^b^ | Gene product |
| --- | --- | --- |
| Down-regulated (26 genes) |  |  |
| VVMO6_RS15985 | -8.58 | Diacylglycerol kinase DgkA |
| VVMO6_RS21740 | -7.60 | TolC-like outer membrane channel protein TolCV2 |
| VVMO6_RS15975 | -7.54 | Phosphoglycerol transferase I MdoB |
| VVMO6_RS15980 | -7.24 | Phosphoethanolamine transferase EptA |
| VVMO6_RS21735 | -6.98 | ABC transporter permease |
| VVMO6_RS21745 | -6.84 | Efflux RND transporter periplasmic adaptor subunit |
| VVMO6_RS15995 | -6.27 | Response regulator CarR |
| VVMO6_RS21725 | -5.64 | ABC transporter ATP-binding protein |
| VVMO6_RS18355 | -5.46 | Porin family protein |
| VVMO6_RS21730 | -5.30 | ABC transporter permease |
| VVMO6_RS15880 | -4.79 | Hypothetical protein |
| VVMO6_RS19105 | -4.50 | Hypothetical protein |
| VVMO6_RS21510 | -4.14 | DUF2799 domain-containing protein |
| VVMO6_RS11890 | -4.11 | DNA-directed RNA polymerase subunit beta |
| VVMO6_RS15990 | -2.58 | Sensor kinase CarS |
| VVMO6_RS20180 | -1.68 | Ribosome recycling factor family protein |
| VVMO6_RS20185 | -1.64 | Hypothetical protein |
| VVMO6_RS03680 | -1.58 | DUF3413 domain-containing protein |
| VVMO6_RS06925 | -1.58 | Hypothetical protein |
| VVMO6_RS08860 | -1.56 | DNA transformation protein |
| VVMO6_RS05730 | -1.21 | ABC transporter substrate-binding protein |
| VVMO6_RS17975 | -1.19 | Hypothetical protein |
| VVMO6_RS16585 | -1.16 | Methyl-accepting chemotaxis protein |
| VVMO6_RS09805 | -1.15 | Cytochrome c-type protein NrfB |
| VVMO6_RS12285 | -1.11 | Isocitrate lyase |
| VVMO6_RS05040 | -1.06 | YeaH/YhbH family protein |
| Up-regulated (2 genes) |  |  |
| VVMO6_RS00375 | 1.32 | Hypothetical protein |
| VVMO6_RS03315 | 1.02 | U32 family peptidase |

^a^Locus tags are based on the database for the *V. vulnificus* MO6-24/O genome (GenBank^TM^ assembly accession number GCF_000186585.1).

^b^The fold change represents the ratio of the RPKM of each gene in the *carR* mutant versus the wild type.

**Table S2. Bacterial strains and plasmids used in this study**

| Strain or plasmid | Relevant characteristics^a^ | Reference or source |
| --- | --- | --- |
| Bacterial strains |  |  |
| *V. vulnificus* |  |  |
| MO6-24/O | Wild type; clinical isolate | Laboratory collection |
| TY03209 | MO6-24/O with Δ*carR* | This study |
| TY03206 | MO6-24/O with Δ*eptA* | This study |
| TY21083 | MO6-24/O with Δ*dgkA* Δ*eptA* Δ*mdoB* | This study |
| DH226 | MO6-24/O with ΔVVMO6_RS21745-RS21725 | This study |
| TY03208 | MO6-24/O with Δ*carS* | This study |
| GR233 | MO6-24/O with *carR*_D55A_ | This study |
| GR234 | MO6-24/O with *carS*_H220A_ | This study |
| *E. coli* |  |  |
| S17-1 λ*pir* | λ-*pir* lysogen; *thi pro hsdR hsdM*^+^ *recA* RP4-2 Tc::Mu-Km::Tn7;Tp^r^ Sm^r^; host for π-requiring plasmids | (2) |
| BL21(DE3) | *F^-^*, *ompT*, *hsdS* (r_B_^-^, m_B_^-^), *gal dcm* (DE3) | Laboratory collection |
| Plasmids |  |  |
| pDM4 | Suicide vector; R6K γ *ori* *sacB*; *oriT* of RP4; Cm^r^ | (3) |
| pTY03209 | pDM4 with Δ*carR*; Cm^r^ | This study |
| pTY03207 | pDM4 with Δ*dgkA*; Cm^r^ | This study |
| pTY03206 | pDM4 with Δ*eptA*; Cm^r^ | This study |
| pTY03205 | pDM4 with Δ*mdoB*; Cm^r^ | This study |
| pDH2210 | pDM4 with ΔVVMO6_RS21745-RS21725; Cm^r^ | This study |
| pTY03208 | pDM4 with Δ*carS*; Cm^r^ | This study |
| pGR2101 | pDM4 with *carR*_­D55A_ | This study |
| pGR2102 | pDM4 with *carS*_­H220A_ | This study |
| pJK1113 | pKS1101 with *nptI*; Ap^r^ Km^r^ | (4) |
| pTY21091 | pJK1113 with *carR*; Ap^r^ Km^r^ | This study |
| pET-28a(+) | His_6_-tagged fusion protein expression vector; Km^r^ | Novagen |
| pTY21092 | pET-28(a)+ with *carR*; Km^r^ | This study |
| pDH2214 | pET-28(a)+ with *carR*_D55A_; Km^r^ | This study |
| pBBR-lux | Broad-host-range vector; promoterless *luxCDABE* operon; Cm^r^ | (5) |
| pDH2207 | pBBR-lux with 371–bp fragment of *carR* upstream region; Cm^r^ | This study |

^a^Tp^r^, trimethoprim-resistant; Sm^r^, streptomycin-resistant; Cm^r^, chloramphenicol-resistant; Ap^r^, ampicillin-resistant; Km^r^, kanamycin-resistant.

**Table S3. Oligonucleotides used in this study**

| **Oligonucleotide** | **Oligonucleotide sequence, 5’** **→3’^a, b^** | **Use** |
| --- | --- | --- |
| **For mutant construction** | | |
| CARRD-F1 | GAGCTCAGGTTACCCGCATGCTTCAATTGACCAAGCAC | Deletion of *carR* ORF |
| CARRD-R1 | TTGTCAATGGGGATCCCGGTGATCACTGAAAATC |  |
| CARRD-F2 | GTGATCACCGGGATCCCCATTGACAATCGTGTGG |  |
| CARRD-R2 | CGACCCTCGAGTACGCGTCAGCTCGTAGATGGTTAAAATAG |  |
| DGKAD-F1 | GAGCTCAGGTTACCCGCATGGGCATCATTCACCTTACC | Deletion of *dgkA* ORF |
| DGKAD-R1 | TCGCCCAAACGGATCCGTCGACAATACGACGAATAC |  |
| DGKAD-F2 | TATTGTCGACGGATCCGTTTGGGCGAGTGTATTG |  |
| DGKAD-R2 | CGACCCTCGAGTACGCGTCACATGATGTTGGCAATCATG |  |
| EPTAD-F1 | GAGCTCAGGTTACCCGCATGATGAATCGAATCAAAACCTTG | Deletion of *eptA* ORF |
| EPTAD-R1 | TCACCAAGAGGGATCCCATAGCTTACCATCGCAG |  |
| EPTAD-F2 | GTAAGCTATGGGATCCCTCTTGGTGAGAATGGTTTG |  |
| EPTAD-R2 | CGACCCTCGAGTACGCGTCACATTGCTTAAAGAGATCAAGG |  |
| MDOBD-F1 | GAGCTCAGGTTACCCGCATGATGCGTAAGATTAATTTAGGTCC | Deletion of *mdoB* ORF |
| MDOBD-R1 | TGTTGTTCGCGGATCCCATGATCCAAAGACCAGC |  |
| MDOBD-F2 | TTGGATCATGGGATCCGCGAACAACATCGATATG |  |
| MDOBD-R2 | CGACCCTCGAGTACGCGTCATTAATAATCCGATTTGTACCAATTG |  |
| RS21745D-F2 | AGCTCAGGTTACCCGCATGCGGAATCATAATGACGAAACG | Deletion of *tolCV2* operon |
| RS21745D-R2 | TAGGGGATCCTTTTTGCTTGGCGGATTCGA |  |
| RS21725D-F2 | CAAAAAGGATCCCCTACAGGGGCATTGGATTC |  |
| RS21725D-R2 | CTCGAGTACGCGTCACTAGTTTAGAGCACTCGGGTATCGC |  |
| CARSD-F1 | GAGCTCAGGTTACCCGCATGCCGATCAAACCATCAATTTAAC | Deletion of *carS* ORF |
| CARSD-R1 | GACAAATCTGGGATCCCAGTACACGATCGTAGTC |  |
| CARSD-F2 | TCGTGTACTGGGATCCCAGATTTGTCAGATTCACAAC |  |
| CARSD-R2 | CGACCCTCGAGTACGCGTCAGTCGCTAAGCTACTTCAAC |  |
| CARRPM-R1 | GGTAACATCAGAGCAAGCAGGACGAGTG | Point mutation of *carR* ORF |
| CARRPM-F2 | CTGCTTGCTCTGATGTTACCTGGTCAAGATG |  |
| CARSPM-R1 | GCGTAGCTCAGCAGCCACAGCATTAG | Point mutation of *carS* ORF |
| CARSPM-F2 | CTGTGGCTGCTGAGCTACGCACAC |  |
| **For mutant complementation** | | |
| CARRC-F | GCTAGCAGGAGGAATTCACCATGGTAGAAGACCAGAAG | Amplification of *carR* ORF |
| CARRC-R | AAAACAGCCAAGCTTGCATGTTTCGGTGTTTATTCATTCC |  |
| **For qRT-PCR** | | |
| EPTA_qRT-F | TGCCTTACAGTTTAGCCCCG | Quantification of *eptA* expression |
| EPTA_qRT-R | GTCGCATCGTACTCACTGGT |  |
| TOLCV2_qRT-F | AAGCTGGATGAGGGCAACAA | Quantification of *tolCV2* expression |
| TOLCV2_qRT-R | TCCGCTTGGGCATTGAGAAT |  |
| CARR_qRT-F | CCATTGACAATCGTGTGGTC | Quantification of *carR* expression |
| CARR_qRT-R | TTATTCATTCCATGTGTCTGGC |  |
| **For protein overexpression** | | |
| CARRP-F | CTTTAAGAAGGAGATATACCATGGTAGAAGACCAGAAG | Amplification of *carR* and *carR*_D55A_ ORF |
| CARRP-R | CAGTGGTGGTGGTGGTGGTGTTCATTCCATGTGTCTGG |  |
| **For EMSA and DNase I protection assay** | | |
| PEPTA-F | GTGCCTGCTTCATCATTCGTTTTTCCC | Amplification of *eptA* upstream region |
| PEPTA-R | GCGGTGATGGTTAGCGCG |  |
| PCARR-F | CGAACCAGTACCTCAATCAGC | Amplification of *carR* upstream region |
| PCARR-R | CATCTGTTGGAGCTTGATGTCATC |  |
| **For reporter construction** | | |
| PcarRX-F | GGGCGAATTGGAGCTCCAGTACCTCAATCAGCGCAT | Amplification of *carR* upstream region |
| PcarRX-R | CCGCAACTAGAGGATCCACATCTGTTGGAGCTTGATGTCATC |  |

^a^The oligonucleotides were designed using the *V. vulnificus* MO6-24/O genomic sequence (GenBank^TM^ assembly accession number GCF_000186585.1).

^b^Regions of oligonucleotides not complementary to the corresponding genes are underlined.

# References

1. Notredame C, Higgins DG, Heringa J. 2000. T-Coffee: A novel method for fast and accurate multiple sequence alignment. Journal of Molecular Biology 302:205-217.

2. Simon R, Priefer U, Puhler A. 1983. A Broad Host Range Mobilization System for *In vivo* Genetic Engineering: Transposon Mutagenesis in Gram Negative Bacteria. Bio-Technology 1:784-791.

3. Milton DL, OToole R, Horstedt P, WolfWatz H. 1996. Flagellin A is essential for the virulence of *Vibrio anguillarum*. Journal of Bacteriology 178:1310-1319.

4. Lim JG, Bang YJ, Choi SH. 2014. Characterization of the *Vibrio vulnificus* 1-Cys Peroxiredoxin Prx3 and Regulation of Its Expression by the Fe-S Cluster Regulator IscR in Response to Oxidative Stress and Iron Starvation. Journal of Biological Chemistry 289:36263-36274.

5. Lenz DH, Mok KC, Lilley BN, Kulkarni RV, Wingreen NS, Bassler BL. 2004. The small RNA chaperone Hfq and multiple small RNAs control quorum sensing in *Vibrio harveyi* and *Vibrio cholerae*. Cell 118:69-82.
